# Empowering parents of children with ADHD through artificial intelligence

**DOI:** 10.1186/s13034-025-00933-1

**Published:** 2025-09-30

**Authors:** Aaradhana Rukadikar, Komal Khandelwal

**Affiliations:** Symbiosis International (Deemed University), Pune, India

A compelling and contemporary analysis of how digital information influences parental decision-making and caregiving journeys is provided by the recently published study that examines parents’ lived experiences of looking for information about attention deficit hyperactivity disorder (ADHD) online. This study highlights that although the internet frequently acts as a lifeline, providing access to communities, information, and emotional support, it is also a source of false information, conflicting stories, and cognitive overload [1]. In this context, this correspondence provides a supplementary perspective on how technology, namely artificial intelligence (AI), might revolutionize the online ecosystem of mental health information and better assist parents in overcoming the difficulties associated with parenting children with ADHD.

## Implementing AI for ADHD assistance

The following are the suggestions for implementing AI in a revolutionary and decisive way in this complex and emotionally charged environment.

### Trustworthy information

In contrast to a simple keyword-based search engine, AI algorithms may be trained to produce and select excellent, validated, and evidence-based material on the user history of parents in terms of recent searches, context, and special concerns to provide verified and personalized content. Similarly, AI can summarize academic studies, medical research, medical literature, and simplified explanations of medical recommendations [2]. By providing clear expectations of data use, opt-in choices for tailored content, encrypted data, and user control over recommendations; ethics can be ensured in the personalization of the treatment.

### Peer support

AI-based peer support tools are digital platforms that enhances social and emotional support such as *little otter*,* talk2us.ai*,* and careclinic.io* can be used to highlight evidence-based community responses, flag inaccurate information about ADHD, identify and eliminate harmful stigmatizing language, suggest experience-verified resources, and summarize recurring themes on the basis of strategies that others have found helpful [3].

### Mediator between parents and health professionals

Following a clinical visit, medical providers may provide parents with AI-assisted frequently asked questions (FAQ) platforms that are curated with feedback from specialists in lived experience and medicine [4].

## Innovative ways to strengthen AI assistance

### Decision support systems

Parents can be guided through a series of structured questions by AI-powered decision support systems, which can then provide tailored recommendations on the basis of parental preferences and current clinical guidelines. To promote more meaningful and knowledgeable relationships with healthcare providers, these systems can serve as powerful tools. AI-based decision systems must be trained on datasets that reflect current diagnostic criteria and treatment pathways outlined by official clinical guidelines embedded within the clinical workflow, with explainable outputs.

### Real-time support

AI-powered chatbots can listen to and sympathetically address parental worries, provide evidence-based answers to questions about ADHD, provide techniques for regulating emotions, and, if necessary, link users to communities for round-the-clock assistance.

### Ensuring ethical, inclusive, and equitable AI solutions

AI systems must be developed ethically and inclusively [5].

Ethical: AI systems that incorporate a variety of datasets and consider cultural sensitivity into consideration while accounting for differences in caring behaviors and stigma.

Inclusive: To bridge the digital literacy gap, interfaces must be voice-assisted, multilingual, and easy to use for parents who are not tech-savvy.

Equitable: AI systems must protect data privacy and confidentiality since mental health and child-related data are sensitive.

AI systems must be carefully informed, accessible, sustainable, and transparent. Only then can it be harnessed with full potential to support families in navigating the challenges of ADHD without compromising their dignity, privacy, or autonomy. By embedding ethics from conception to implementation, we can develop AI systems that not only assist but also empower those who care for children with ADHD.

The moment has come to develop and deploy human-centered AI systems that help parents by interpret data, help, and fill in gaps in the mental health of children with ADHD via a holistic approach.

## Data Availability

No datasets were generated or analysed during the current study.

## Abbreviations

ADHD: Attention deficit hyperactivity disorder
AI: Artificial intelligence
FAQ: Frequently asked questions
